# Does case misclassification threaten the validity of studies investigating the relationship between neck manipulation and vertebral artery dissection stroke? No

**DOI:** 10.1186/s12998-016-0124-9

**Published:** 2016-11-05

**Authors:** Donald R. Murphy, Michael J. Schneider, Stephen M. Perle, Christopher G. Bise, Michael Timko, Mitchell Haas

**Affiliations:** 1 Care New England Health System, Department of Family Medicine, Alpert Medical School of Brown University, 600 Pawtucket Avenue, Pawtucket, RI 02860 USA; 2 University of Pittsburgh, Bridgeside Point 1, 100 Technology Drive, Suite 210, Pittsburgh, PA 15219-3130 USA; 3 University of Bridgeport, Bridgeport, CT 06604 USA; 4 School of Health and Rehabilitation Science, University of Pittsburgh, Bridgeside Point 1, 100 Technology Drive, Suite 210, Pittsburgh, PA 15219-3130 USA; 5 Department of Physical Therapy, University of Pittsburgh, Bridgeside Point Suite 228, 100 Technology Drive, Pittsburgh, PA 15219-3130 USA; 6 University of Western States, 2900 NE 132nd Avenue, Portland, OR 97230 USA

**Keywords:** Vertebral artery dissection, Cervical manipulation, Stroke, Complications to manipulation, Chiropractic, Manual therapy

## Abstract

**Background:**

The purported relationship between cervical manipulative therapy (CMT) and stroke related to vertebral artery dissection (VAD) has been debated for several decades. A large number of publications, from case reports to case–control studies, have investigated this relationship. A recent article suggested that case misclassification in the case–control studies on this topic resulted in biased odds ratios in those studies.

**Discussion:**

Given its rarity, the best epidemiologic research design for investigating the relationship between CMT and VAD is the case–control study. The addition of a case-crossover aspect further strengthens the scientific rigor of such studies by reducing bias. The most recent studies investigating the relationship between CMT and VAD indicate that the relationship is not causal. In fact, a comparable relationship between vertebral artery-related stroke and visits to a primary care physician has been observed. The statistical association between visits to chiropractors and VAD can best be explained as resulting from a patient with early manifestation of VAD (neck pain with or without headache) seeking the services of a chiropractor for relief of this pain. Sometime after the visit the patient experiences VAD-related stroke that would have occurred regardless of the care received.

This explanation has been challenged by a recent article putting forth the argument that case misclassification is likely to have biased the odds ratios of the case–control studies that have investigated the association between CMT and vertebral artery related stroke. The challenge particularly focused on one of the case–control studies, which had concluded that the association between CMT and vertebral artery related stroke was not causal.

It was suggested by the authors of the recent article that misclassification led to an underestimation of risk. We argue that the information presented in that article does not support the authors’ claim for a variety of reasons, including the fact that the assumptions upon which their analysis is based lack substantiation and the fact that any possible misclassification would not have changed the conclusion of the study in question.

**Conclusion:**

Current evidence does not support the notion that misclassification threatens the validity of recent case–control studies investigating the relationship between CMT and VAD. Hence, the recent re-analysis cannot refute the conclusion from previous studies that CMT is not a cause of VAD.

## Background

Cervical artery dissection (CAD) is a condition in which a tear forms in the wall of one of the cervical arteries, leading to partial or complete occlusion of the arterial lumen. A thrombus can form as a result of this tear, with subsequent embolization, leading to stroke somewhere within the arterial system in which the condition began. The general condition known as CAD can involve either the vertebral artery (vertebral artery dissection – VAD) or the internal carotid artery (internal carotid artery dissection – ICAD). VAD and ICAD are similar but have distinctly identifiable clinical features that differentiate them [1].

An observed association between cervical manipulative therapy (CMT) and stroke related to CAD has been reported for several decades [2]. The greatest scrutiny has involved the relationship between CMT and VAD. This relationship has been studied with increasingly rigorous methodology over that time.

Early in the evolution of the study of the relationship between CMT and VAD, a cause-effect relationship was assumed because several cases were reported in which an individual experienced VAD after receiving CMT. This is a common logical fallacy known in formal logic as *post hoc ergo propter hoc* (from the Latin meaning: “after this, therefore because of this”). Correlation, however, is not synonymous with causation. Causal inference requires a research design employing a comparison group. For the study of rare clinical events, such as the relation between CMT and VAD, prospective cohort studies are not feasible, so the best evidence must come from appropriately designed and properly conducted case–control studies [3, 4].

In case–control studies, case misclassification of various types can be a potential confounding factor. Thus, it is important that researchers conducting case–control studies appropriately design their studies to minimize case misclassification, to confirm, if possible, that misclassification has not occurred and, most important, to determine whether misclassification would alter the investigators’ conclusions.

## Discussion

As study of the relationship between CMT and VAD evolved, case–control studies confirmed a statistical association between CMT and stroke related to the vertebral artery (but not the internal carotid artery). The first of these studies was that of Rothwell et al. [5] which compared 582 individuals with vertebral artery-related stroke (cases) with 2328 matched individuals with no history of stroke (controls). They found that cases under age 45 were five times more likely to have had a visit to a chiropractor within one week of their stroke. In individuals 45 or older, there was no difference between cases and controls.

Later, Smith et al. [6] compared 25 patients who had experienced strokes that were attributed to VAD and 100 patients with other types of stroke. They asked the patients to recall over the previous 30 days whether they had seen a chiropractor. They found that patients diagnosed with VAD were six times more likely than controls to recall having seen a chiropractor within 30 days of having the stroke. Smith et al. [6] did not find any association between CMT and stroke related to ICAD. This is consistent with other studies that have found a lack of association between visits to practitioners of CMT and stroke when the conditions of VAD and ICAD are examined together [7, 8].

While these early case–control studies suggested a statistical association between CMT and VAD (but not between CMT and ICAD), it could still not be determined whether the relationship between CMT and VAD is correlational, causal or chance. Further, even if the relationship were causal, these study designs were not able to distinguish which is “cause” and which is “effect”. In other words, the question remained as to whether:

1. CMT is a rare cause of VAD or;
2. The common early symptoms of VAD (in over 2/3 of patients neck pain or headache [9]) can lead a patient to seek the services of a practitioner of CMT, with the stroke occurring sometime after, independent of the CMT.

In an attempt to answer this question, Cassidy et al. [10] conducted a case–control study, with the addition of a case-crossover design for the purpose of reducing bias. In this study, 818 patients with vertebral artery-related stroke (cases) were compared to 3164 matched controls with no history of vertebral artery-related stroke. Most importantly, the authors included not only visits to chiropractors, but also visits to primary care physicians (PCPs) so that they could compare the association between chiropractic visits and vertebral artery-related stroke and between PCP visits and vertebral artery-related stroke.

As with the Rothwell et al. study [5], Cassidy et al. [10] found an association between visits to chiropractors and vertebral artery-related stroke in patients under age 45, but not in patients age 45 and older. They also found an association between visits to PCPs and vertebral artery-related stroke, both in patients under age 45 and in patients age 45 and older. Further, the association of visits both to chiropractors and to PCPs with vertebral artery-related stroke was greater when the visits involved neck pain or headache.

These data suggest that the most plausible explanation for the association between visits to chiropractors and VAD is that CMT is not a cause of VAD [11], but rather that patients who experience neck pain and/or headache resulting from the arterial dissection seek the services of a practitioner of CMT for relief of this pain, then go on to develop a stroke after this visit, independent of the CMT. This would also explain the similar associations of stroke between chiropractic and PCP patients; some patients with these symptoms will see their PCP and others a chiropractor.

Thus, earlier assumptions in case reports and case–control studies that CMT was the cause of VAD can be attributed to protopathic bias. Protopathic bias is common in case–control studies, as well as case reports and case series, and occurs when a therapeutic agent is applied for the early symptoms of an occult disease and, when the patient later develops the full manifestation of the disease, the therapeutic agent is mistakenly assumed to have been its “cause” [12].

Further evidence in support of this explanation was provided in a recent case–control study by Kosloff et al. [13]. This study used claims data of commercially insured and Medicare Advantage health plan members and compared 1829 patients with vertebral artery-related stroke with 4633 healthy, matched controls. They found no association between vertebral artery-related stroke and visits to chiropractors. They did find an association between patients with vertebral artery-related stroke and visits to PCPs.

Importantly, they also found that, among those patients who experienced vertebral artery-related stroke after seeing a chiropractor, in one-third of visits in the commercial population and half of visits in the Medicare Advantage population *no CMT was applied at the time of these visits*. This further supports number two listed above as the most plausible explanation for the association between visits to chiropractors and vertebral artery-related stroke. If no CMT was applied with these patients, CMT could not possibly have caused their strokes.

The only study we were able to find that has suggested that misclassification might threaten the validity of studies investigating the relationship between CMT and stroke related to VAD is that of Cai et al. [14]. Therefore, much of our discussion will focus on that study.

Cai et al. [14] attempted to investigate whether case misclassification had an impact on the odds ratios of the Rothwell et al. [5] and Cassidy et al. [10] studies regarding the association between manipulation and cervical artery [sic] dissection. These authors suggested that case misclassification likely occurred in those studies because of the use of diagnostic codes related to the location of the stroke (the posterior circulation) rather than the mechanism that led to the stroke (arterial dissection).

It is notable that, unlike Rothwell et al. [5] and Cassidy et al. [10], Cai et al. [14] included both vertebral artery-related stroke and internal carotid artery-related stroke in their analysis and did not distinguish between these conditions. This is important for two reasons. First, these conditions have important clinical features that distinguish them [1] and, second, case–control studies have not found an association between CMT and ICAD [6, 7, 15].

Cai, et al. [14] used data from the Veterans Health Administration (VA) electronic medical record and searched for eight ICD-9 codes related to stroke involving either the vertebral or the internal carotid arteries. They pointed out that they included three codes that identify arterial dissection that were not available at the time of the Rothwell et al. [5] and Cassidy et al. [10] studies. Cai et al. [14] concluded that, based on their analysis, “Prior studies grossly misclassified cases of cervical artery dissection and mistakenly dismissed a causal association with manipulation.” They further go on to state, “Our study indicates that the [odds ratios] for spinal manipulation exposure in cervical artery dissection is higher than previously reported.”

There are numerous erroneous statements and methodological flaws related to the Cai et al. [14] paper that undermine its usefulness to the discussion of both causation and misclassification as they apply to CMT and VAD. We will only focus here are those issues specifically related to the question of misclassification and comparison to studies related to VAD:

1. The Results section of the Abstract states “we reanalyzed a previous study, which reported *no association* [emphasis added] between spinal manipulation and cervical artery dissection…” The only study that was reported in the paper to have been “reanalyzed” was that of Cassidy et al. [10]. The Cassidy et al. study [10] did not investigate any association between spinal manipulation and “cervical artery dissection” (which, as stated earlier, includes both VAD and ICAD). It only investigated the association between spinal manipulation and stroke related to the *vertebral artery*. Therefore, no reasonable comparison can be made between the Cai et al. [14] paper and that of Cassidy et al. [10].
2. Furthermore, contrary to the statement quoted above by Cai et al. [14], the Cassidy et al. study [10] did report a statistical association between visits to chiropractors and stroke related to the vertebral artery, at least in individuals under 45 years of age. Statistical association was also found between visits to PCPs and stroke related to the vertebral artery.
3. Statistical analysis always requires assumptions to be made and the accuracy of the results depends on the validity of those assumptions. A critical assumption made by Cai et al. [14] was that the misclassification rates (and hence the positive predictive values) of stroke were different for patients who saw chiropractors and those who did not. These differences appear to be based, at least in part, on the assumption that primary care physicians are more likely to see patients with strokes secondary to arthrosclerosis, and hence, have more misclassification errors.

Because Cai et al. [14] chose a smaller stroke misclassification rate for patients *with* a chiropractic visit than for patients *without* a chiropractic visit, the odds ratio increased and led to their presumptive conclusion that Cassidy et al. [10] underestimated the risk of stroke associated with CMT. Had the authors chosen equal misclassification rates, there would have been no effect on the odds ratio as found by Cassidy et al. [10]; and had the authors chosen a larger misclassification rate for chiropractic patients, they would have had to conclude that Cassidy et al. [10] *overestimated* the risk of stroke associated with CMT. A sensible course of action by Cai et al. [14] would have been to conduct a broader sensitivity analysis to explore how different assumptions regarding potential sources of misclassification could affect the relative misclassification rates and alter the authors’ results (odds ratios) and conclusions.

4. In the Conclusion section of their abstract, Cai et al. [14] state, “Prior studies grossly misclassified cases of cervical dissection and *mistakenly dismissed a causal association with manipulation* [emphasis added].” In the absence of relevant epidemiological evidence that “gross misclassification” occurred in these prior studies, the authors cannot challenge the similarity of ORs for stroke risk in chiropractic and PCP patients, evidence that disputes a potential causal relationship between manipulation and stroke.
5. If misclassification were to have occurred in Cassidy et al. [10], it would have affected the odds ratios for stroke risk for PCP and chiropractic exposures equally. Again, the significant association between vertebral artery-related stroke and exposure to both chiropractors and PCPs remain similar and does not change the conclusion regarding a lack of causal relation between visits to chiropractors and stroke.
6. In the Cai et al. [14] study, less than 4 % of subjects were under age 45. VAD is generally a condition of younger people [1], with the incidence of VAD in older people being extremely low [16]. Therefore, this significantly limits the generalizability of the study as well as the ability to compare this study with that of Cassidy et al. [10] or, for that matter, any study related to VAD.
7. Further lack of generalizability and direct comparison comes from the fact that the Cai et al. [14] study used a patient population (VA patients) that was distinctly different from that of Cassidy et al. [10] (the general public in the province of Ontario).
8. Cai et al. [14] did not report the percentage of subjects in their cohort who were male and female, but they indicate that less than 10 % of the VA patient population is female. Females made up 37 % of the cases in the Cassidy, et al. [10] study, 39 % of the cases in the Rothwell et al. [5] study and 33 % of the cases in the Smith et al. [6] study. In other studies, females have made up 50 % or more of cases of VAD [17]. This further limits the generalizability of their data as well as the comparison to studies related to VAD.

Cassidy et al. [10] did investigate whether possible case misclassification impacted their findings and conclusions through the use of sensitivity analysis. Sensitivity analysis did have an impact on the calculation of their odds ratios. However, the most important point is that it did not impact the main conclusions of the paper because any possible misclassification would have impacted both comparisons equally (chiropractor and PCP). Thus, the comparison between visits to chiropractors in patients with vertebral artery-related stroke and visits to PCPs in these patients would remain the same.

Furthermore, the database that Cassidy et al. [8] used has been independently studied for validity. It was found that agreement with the original stroke diagnosis occurred in 85.4 % (95 % CI 83 % to 88 %) of cases [18]. This suggests that rate of misclassification is substantially less than estimated by Cai et al. [14].

Given the rarity of VAD, case–control studies using large administrative datasets are the best research design in investigating the relationship between this disorder and any exposure, including CMT. However, there are some inherent challenges with case classification when using only diagnosis and procedure codes obtained from administrative data. Efforts to minimize the degree of case misclassification, and any impact this may have on the study’s conclusions, are important. We suggest that three major issues need to be addressed in order to minimize case misclassification:

1. Inappropriate pooling of vertebral artery dissection with internal carotid artery dissection, as well as inappropriate pooling of strokes related to dissection with strokes related to other causes, such as atherosclerosis, arteriovenous malformation or aneurism. Investigating this question of misclassification should rely on case–control studies that clearly identify those cases that involve *vertebral artery dissection* as the reported diagnosis. When large administrative datasets are used, the ICD 9 code (443.24) or the ICD 10 code (I77.74) for VAD should be included in the search. This will maximize the likelihood that patients with strokes related to vertebral artery dissection are identified as “cases”.
2. Inappropriate assumptions that any “chiropractic visit” involves CMT, i.e., it is important to identify whether manipulation was performed on the chiropractic visits in question, as was done by Kosloff et al. [13]. This requires identification of the CPT code group 98940 through 98942 when gathering data.
3. Control for what Church et al. [11] refer to as “interviewer bias.” Because of the highly publicized nature of many case reports and anecdotes about the relationship between visits to practitioners of CMT and stroke, emergency department physicians and neurologists who see patients with posterior circulation stroke may be more likely to attribute such a stroke to VAD resulting from CMT. Thus, many cases may be erroneously coded as VAD in the administrative database. Misdiagnosis of stroke is common in emergency department settings [19].

This phenomenon is further impacted by the fact that, as compared to other practitioners, neurologists are likely to be made aware of a far greater number of patients who have had VAD after having seen a chiropractor [20]. This is particularly impactful in cases in which, due to *post hoc ergo propter hoc* and protopathic bias, the visit to the chiropractor was incorrectly assumed to have “caused” the stroke. Interviewer bias can lead to a greatly increased likelihood that any case of posterior circulation stroke will be attributed to VAD that was “caused” by CMT, whether the clinical facts support this conclusion or not. And, again, this will lead to a greatly increased likelihood that the diagnostic code for VAD will be inappropriately entered into the administrative database. Therefore, interviewer bias must be considered in any investigation of CMT and stroke

## Conclusion

Current evidence from case–control studies indicates that the statistical association between visits to chiropractors and vertebral artery related stroke results from patients with the early symptoms of VAD seeking the services of a practitioner of CMT prior to experiencing the full manifestation of VAD-related stroke. Any assumption that CMT is a cause of VAD can be attributed to protopathic bias.

In the case of the relationship between CMT and VAD, there is no current compelling evidence that case misclassification in the case–control studies in this area would alter the conclusion, based on data from case–control studies, that the relationship between CMT and VAD is not causal.

Case–control studies using large administrative databases are an efficient way to investigate any association between CMT and VAD. Inherent in the use of administrative data is the possibility of misclassification. This misclassification can come in the form of inappropriate pooling of VAD and ICAD, inappropriate pooling of stroke caused by arterial dissection with stroke resulting from other arterial pathologies, inaccurate use of ICD-9 or ICD-10 codes, inappropriate assumption that any chiropractic visit includes CMT, protopathic bias and interviewer bias. It is important that scientists who conduct case–control studies utilize sensitivity analysis as well as other means to minimize misclassification and to determine whether misclassification may impact the conclusions they draw from their data.

## Abbreviations

CAD: Cervical artery dissection
CMT: Cervical manipulative therapy
ICAD: Internal carotid artery dissection
PCPs: Primary care physicians
VA: Veterans Health Administration
VAD: Vertebral artery dissection
